# Insights into the Evolution of the CSP Gene Family through the Integration of Evolutionary Analysis and Comparative Protein Modeling

**DOI:** 10.1371/journal.pone.0063688

**Published:** 2013-05-28

**Authors:** Jonna Kulmuni, Heli Havukainen

**Affiliations:** 1 Department of Biology and Biocenter Oulu, University of Oulu, Oulu, Finland; 2 Department of Biosciences, Centre of Excellence in Biological Interactions, University of Helsinki, Helsinki, Finland; 3 Department of Chemistry, Biotechnology and Food Science, Norwegian University of Life Sciences, Aas, Norway; 4 School of Life Sciences, Arizona State University, Tempe, Arizona, United States of America; University of Georgia, United States of America

## Abstract

Insect chemical communication and chemosensory systems rely on proteins coded by several gene families. Here, we have combined protein modeling with evolutionary analysis in order to study the evolution and structure of chemosensory proteins (CSPs) within arthropods and, more specifically, in ants by using the data available from sequenced genomes. Ants and other social insects are especially interesting model systems for the study of chemosensation, as they communicate in a highly complex social context and much of their communication relies on chemicals. Our ant protein models show how this complexity has shaped CSP evolution; the proteins are highly modifiable by their size, surface charge and binding pocket. Based on these findings, we divide ant CSPs into three groups: typical insect CSPs, an ancient 5-helical CSP and hymenopteran CSPs with a small binding pocket, and suggest that these groups likely serve different functions. The hymenopteran CSPs have duplicated repeatedly in individual ant lineages. In these CSPs, positive selection has driven surface charge changes, an observation which has possible implications for the interaction between CSPs and ligands or odorant receptors. Our phylogenetic analysis shows that within the Arthropoda the only highly conserved gene is the ancient 5-helical CSP, which is likely involved in an essential ubiquitous function rather than chemosensation. During insect evolution, the 6-helical CSPs have diverged and perform chemosensory functions among others. Our results contribute to the general knowledge of the structural differences between proteins underlying chemosensation and highlight those protein properties which have been affected by adaptive evolution.

## Introduction

Chemical communication is crucial for insects, as their perception of the world is dominated by odors. Most of their behavior from courtship and mating to locating resources such as food and a suitable habitat are dependent on chemical senses. Insect chemosensory systems have been studied using several approaches, and information on specific genes, neurological processes and biochemical properties of the chemosensory systems is constantly growing. From an evolutionary point of view, chemosensory systems are interesting because of their potential role in adaptation and speciation [1]. Knowledge of insect chemosensation also has practical importance through potential applications for pest control.

The reception of chemical messages in insects starts when specific carrier proteins, such as the odorant binding proteins (OBP) or chemosensory proteins (CSP), bind and solubilize odorants and pheromones and transport them through the aqueous hemolymph [2],[3]. The chemical messages carried by the OBPs and CSPs are decoded when odorant receptors (OR), or in some cases gustatory receptors (GR), selectively bind the chemicals [4]. All chemosensory genes (OBP, CSP, OR and GR) can form large gene families, each containing from a few to several hundreds of genes, depending on species. Chemosensory gene families have been intensively studied in the context of gene family dynamics and they usually show birth-and-death evolution with purifying selection being the main force [5], [6], [7].

Here, we concentrate on CSPs. CSPs are small globular proteins with a hydrophobic binding pocket and usually containing six α-helices. The CSP gene family varies in size across arthropods; the tick *Ixodes scapularis* has one CSP gene, *Drosophila melanogaster* has four, and the largest known repertoires are found in the flour beetle *Tribolium castaneum* (19 genes), the silkworm *Bombyx mori* (22 genes) and the fire ant *Solenopsis invicta* (21 genes) [8], [9]. Even though the number of CSP genes can be similar in two species, few of them form orthologous pairs. Instead, a large proportion of these genes are specific to certain taxonomic lineages, having duplicated independently in each lineage and evolved functions specific to that lineage. While several studies have investigated the evolutionary forces driving the evolution of these chemosensory gene families, relatively little is known about the precise function of specific genes. Most functional information comes from expression studies and some structural studies characterizing the binding properties of CSPs.

While some CSPs bind and transmit chemical messages, others are involved in processes such as development [10] and possibly immune responses [11]. Generally, insect CSPs are highly expressed in the sensillar lymph and, *in vitro*, capable of binding different components of pheromonal blends [12], but not all CSPs are restricted to chemosensory organs. The honey bee, *Apis mellifera*, the closest studied relative of ants, expresses its CSPs in diverse tissues throughout development [13]. Four of the *A. mellifera* CSPs have been studied in detail, and shown to exhibit different binding spectra. AmelCSP3 possibly binds the brood pheromone, which stimulates the workers to take care of the larvae [14], whereas AmelCSP5 has been shown to play a role in development having a maternal-zygotic expression pattern and being involved in integument formation [11]. Several species among Hymenoptera and Lepidoptera have CSPs with antenna-rich expression suggesting chemosensory function [e.g. [15], [16], [17],[18]] and in ants one CSP plays a role in nestmate recognition [3]. Chemosensory functions have also been implicated in *Locusta migratoria*, where several CSPs show antenna-rich expression and some are involved in the behavioral shift from gregarization to solitarization [19], and in tsetse fly *Glossina morsitans morsitans*, where the expression of CSPs suggests their role in host searching behavior [20]. Summarizing, the CSP proteins perform tasks ranging from ontogeny to colony level regulation.

Ants are a model system to study chemical communication as their communication takes place in a highly complex social context, in which chemicals are used for example to maintain colony cohesion and direct altruistic behavior towards nestmates. The currently available ant genomes (seven species from four ant subfamilies) share seven CSP genes descended from an ancestral genome, forming seven orthologous groups of genes (CSP1-7; [8]). Purifying selection has dominated the evolution of these shared genes, and genes orthologous to some of these seven groups can be found in the honey bee [8]. Of the seven shared ant orthologs, the CSP7 protein is involved in binding nestmate recognition cues in *Camponotus japonicus* [3]. Interestingly, the CSP gene family has expanded repeatedly in individual ant lineages, and the expanded copies appear to have descended from the CSP7 gene [8]. These ant-specific expansions show signs of positive selection, suggesting that they have an adaptive role [8]. Furthermore, they have a fast turnover rate with many gene gain and loss events, and their sequence evolution is characterized by a high dN/dS ratio [8]. The fact that these rapidly evolving ant-specific expansions have originated from the nestmate recognition cue binder CSP7 suggests that the expansions in ants may also have roles related to chemical communication.

Structural biology offers powerful tools for interpreting the evolution and diversity of gene families [21]. These tools can help to understand, among others, the sites of conservation and functional variation in the protein sequences. For example, histones have N- and C-terminal sequence variation that is translated into tail domains of varying size and charge [22], harboring important regulatory modifications [23]. Another well-studied example is the nuclear receptor protein family, which binds small hydrophobic ligands similar to those of CSPs, inside a bundle of α-helices [24]. Changes in this binding pocket are critical to the evolution of nuclear receptors; some of them bind a wide range of ligands, while others are more selective depending on their pocket size and shape [24], [25]. In the case of insect CSPs, structural data is available e.g. for two different CSPs of the moth *Mamestra brassicae* [26], [27] and one desert locust CSP [28], but no comparative structural studies exist.

In this paper, we use comparative protein modeling to characterize the diversity of ant CSPs. We identify variation and conservation in CSP size, charge and structural changes in the binding pocket, as these characteristics can provide the keys to understand the functional diversity of CSPs. CSPs interact both with their ligands and ORs. As the protein surface is the area that first interacts with ligands and receptors, changes in the size and the surface charge could mediate interactions between the CSP and its ligand or OR. Changes in the binding pocket could reflect the ligands the CSPs can accommodate. We also assess whether structural information supports the hypothesis that the expansion of the CSP gene family in ants has led to specialization in tasks related to chemical communication. In that case, the proteins coded by the expanded portion of the CSP family should be more similar to CSPs known to function in chemosensation than to those that function in other tasks, such as development. Previously, we identified sites under positive selection in ant CSPs [8]. Here we map these sites to the protein to infer which parts and properties of the protein have been under positive selection during the expansion of the CSP genes within ants. This will help to relate positive selection to the structural and functional features of the proteins. Finally, we use a large-scale phylogeny combined with the modeled protein structures to infer how structurally different CSPs are distributed within the arthropods. This wider phylogenetic context allowed us to explore how the CSP gene family has evolved within a time frame of 700 My.

## Results

### Orthologous CSPs Differ from each Other in Size, Surface Charges and Binding Pocket

**Figure 1:**
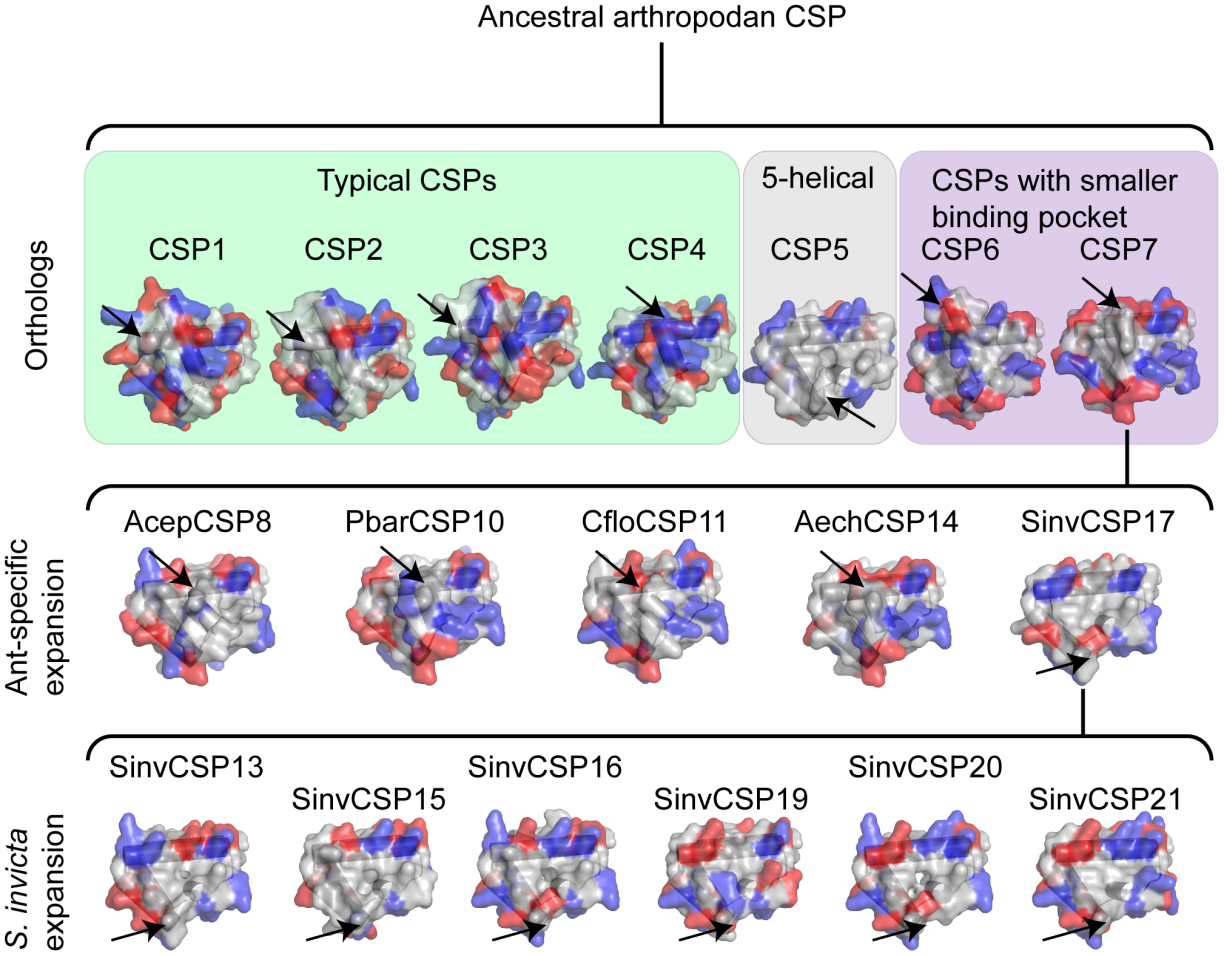
Groups of ant CSPs based on their phylogeny and structural models. All ants share seven orthologous CSPs (CSP1-7; the uppermost row of models), of which CSP7 or a protein similar to that has given rise to the ant-specific expansions (representative proteins on the middle row). The largest currently known ant-expansion is found in *S. invicta* (the lowest row). In the orthologs, CSP1-4 can be grouped together based on their evenly speckled surface charges and similarities in their binding pocket (“Typical CSPs”; green). CSP5 (grey) is conserved across arthropods, and is one of the oldest CSPs. It differs from the other orthologs by having five instead of six helices, a reduced charge on the surface and by changes to its binding pocket. CSP6 and CSP7 are grouped together (purple) due to mutations in their binding pocket that are likely to reduce the size of the pocket cavity. Examples of the ant-specific CSP expansion are shown; *Atta cephalotes* CSP8, *Pogonomyrmex barbatus* CSP10, *Camponotus floridanus* CSP11, *Acromyrmex echinatior* CSP14 and *Solenopsis invicta* CSP17. In the models, negatively charged amino acids (E, D) are shown in red and positively charged amino acids (K, R) in blue. C-termini are marked by an arrow. The *S. invicta* expansion proteins, together with CSP5, are one helix shorter than the other CSPs in their C-terminus, which is depicted by the altered location of the arrow in these models.

We modeled the seven orthologous ant CSPs (CSP1-7) (Figure 1). CSP1-4 are very similar to the template protein (MbraCSPA6, PDB-ID:1KX9) (sequence identity 40–51% at the amino acid level), which makes these models particularly reliable. CSP6 and CSP7 have lower (27% and 32%, respectively) sequence identity compared to the template, but still sufficient to build confident models for the purposes of this study [29]. CSP5 is the least similar (19% identity) of the orthologs, thus, an alternative sequence-based method was used to test the reliability of the model. The sequence-based secondary structure prediction also indicated CSP5 to be a 5-helical protein and located the helices and connective loops almost identically with the model (Dataset S1), supporting the general protein architecture of CSP5 in our model. The quality of the modeled protein structures was further investigated with Ramachandran assessment [30], which was excellent (>95% of residues in favored region and <1% in the outlier region) for CSP1-2, CSP4 and CSP5-6, good for CSP3 (91.5% and 4.7%, respectively) and lower for CSP7 (89.7% of the residues in the favored region and 5.2% outliers).

We compared three characteristics between and among the orthologs: the size, the surface charges and the binding pocket. The size of CSPs appears to be modifiable by adjustments in the length of the last helix (helix 6) (Figure 1, black arrows). In all the seven ant species studied, helix 6 is truncated in CSP4 and CSP7 to approximately half of the size found in most CSPs. The truncation results in a smaller protein size. Ant CSP5 has only five helices. The complete lack of helix 6 results, according to our model, in a wide C-terminal entrance to the binding pocket. This entrance lacks charged amino acid residues and creates a hydrophobic environment atypical of other CSPs (Figure 1).

We next compared the surface charges in CSP1-7. The modeled part of CSP1-7 varied from 89 to 108 amino acids in length, of which 60–70 residues were >20% solvent exposed (Table 1) and here considered surface residues. We identified the surface residues in the sequence alignment (Dataset S2), and recorded any changes in charge (positive, negative or neutral) in the seven ant species for each CSP. The charge variation differs significantly between the CSP1-7 (X^2^ = 61.04, df = 6, P<0.001). Some CSPs appear to have very conserved surface charge; CSP5 has identical surface charge in all seven ant species, and CSP1 has only three surface charge changes (Table 1). This might be a sign of conserved function of these orthologous genes across species. The most prominent charge heterogeneity was found in CSP3 (42.03% of the surface residues had a different charge in at least one species), CSP6 (36.76%) and CSP7 (26.09%), which might be linked to variation in function.

**Table 1:** Surface charge variation in orthologous ant CSPs between the seven ant species.

Orthologous ant CSP	No. >20% solvent exposed aa/number of aas in the model	Number of pos./neg. charged aa on the model surface	No. (and fraction) of solvent exposed aas with charge variation among species
CSP1	70/107	14/17	3 (4.29%)
CSP2	63/106	19/19	12 (19.04%)
CSP3	69/108	19/22	29 (42.03%)
CSP4	63/98	22/17	8 (12.6%)
CSP5	60/89	16/9	0
CSP6	68/104	16/17	25 (36.76%)
CSP7	69/99	14/21	18 (26.09%)

CSP1 and CSP5 have a strictly conserved charge-profile, whereas CSP3, CSP6 and CSP7 show great charge heterogeneity between the ant species. The amino acid (aa) residues with >20% solvent accessible area in the CSP homology models were considered surface residues. The number of positively and negatively charged amino acid residues on the surface of the CSP structure chosen for modeling is shown (represents a single species). The last column shows the number of residues with charge changes in the solvent-exposed residues in all the seven species of ants basing on a sequence alignment.

**Figure 2:**
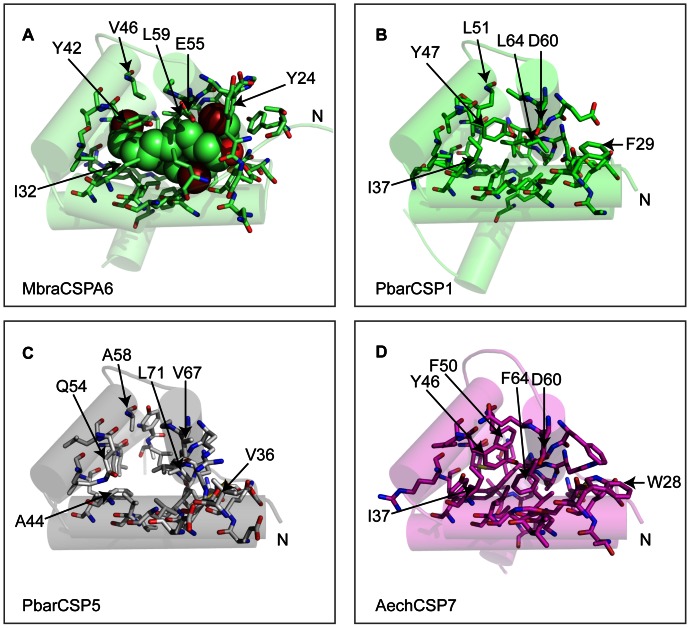
Differences in the size of the CSP binding pockets can reflect ligand differences. The N-terminus is indicated. The 34 binding-residues are shown as sticks. Certain binding-residues of interest are indicated in each CSP. (A) Residues within 5 Å of the ligands in the ligand-bound *M. brassicae* structure (PDB-ID: 1N8V) were considered as binding-residues. The bromo-dodecanol ligands are visualized inside the pocket. (B) The model of CSP1 shown here represents a binding pocket of a non-ligand bound typical CSP protein. (C) The binding pocket of CSP5 is likely enlarged due to the lack of helix 6 and mutations that reduce the size of the binding-residues (A44, Q54, A58 V67 and V36). (D) The binding pocket of CSP7 model is crowded by large binding-residues. For example, F50 and W28 are larger than the corresponding amino acid residues in other, typical CSPs.

As a third characteristic, we studied variation in the binding pocket. Thirty-four amino acid residues in total were defined as ligand-binding residues (i.e. they were at 5 Å distance from the template MbraCSPA6 ligands). These residues border the binding pocket and many, if not most, of them are likely to be involved in ligand binding also in the ant CSPs. Of these 34 binding-residues, six are highly variable in all the ant and other insect CSPs based on the multi-taxa alignment used for modeling (Dataset S2, Table S1). These variable residues were; N67, A82, A86, A87, E106 and R116 (PbarCSP1 numbering). The reasons for this variation remain unclear. They could represent functional variation or, alternatively, residues that have accumulated mutations because of relaxed purifying selection (but see results below). When the two cysteine-bridge forming residues and those located on the last helix (not shared by all CPS) are removed, the number of binding residues decreases to 29. We divided the 29 binding residues into small, intermediate and large based on the side-chain size (Table 2). The binding pockets of ant CSP1-4 appear to be nearly identical, and the size of the binding residues does not differ significantly between any of these CSPs (Chi-squared tests, P>0.05). They largely resemble MbraCSPA6 (Figure 2A–B), and might prefer ligands with similar chemical properties.

**Table 2:** Size distribution of the binding pocket residues.

	CSP1			CSP2		
	Small	intermediate	large	small	intermediate	Large
Sinv	9	17	3	8	16	5
Acep	8	18	3	7	17	5
Aech	8	18	3	8	16	5
Cflo	8	18	3	7	17	5
Hsal	9	17	3	9	15	5
Pbar	9	17	3	8	16	5
Lhum	9	17	3	7	17	5
	**CSP3**			**CSP4**		
	**Small**	**intermediate**	**large**	**small**	**intermediate**	**Large**
Sinv	6	18	5	9	16	4
Acep	3	21	5	8	17	4
Aech	5	19	5	7	18	4
Cflo	7	17	5	8	17	4
Hsal	8	16	5	8	17	4
Pbar	6	18	5	7	18	4
Lhum	6	19	4	7	18	4
	**CSP5**			**CSP6**		
	**small**	**intermediate**	**large**	**small**	**intermediate**	**Large**
Sinv	12	15	2	8	15	5
Acep	12	15	2	8	14	6
Aech	12	15	2	7	15	6
Cflo	12	15	2	9	15	4
Hsal	12	15	2	6	17	5
Pbar	12	15	2	6	17	5
Lhum	12	15	2	8	14	6
	**CSP7**					
	**small**	**intermediate**	**large**			
Sinv	5	12	12			
Acep	6	12	11			
Aech	7	11	11			
Cflo	7	11	11			
Hsal	5	15	9			
Pbar	4	14	11			
Lhum	8	12	9			

Twentynine non-cysteine binding-residues were divided based on their size into small (S,T,C,G,P,A, V) intermediate (H,D,E,N,Q,I,L,M) and large (R, K, F, Y,W) for each CSP in the seven ant species. The binding-residues located in helix-6 were left out due to the lack of helix-6 in CSP5.

The binding pocket of CSP5 is unique; there is a bias towards small-size amino acids, which increases the size of the pocket in our model (Table 2, Table S1, Figure 2C). Using PbarCSP1 as a reference, the size-reducing changes are; F29V, I37A, Y47Q, L51A, D60V, D75G/N/S/D and D110A/V/G; in addition, CSP5 lacks three C-terminal binding residues (where applicable, the residues are indicated in Figure 2). These changes, together with the lack of the last helix, have possibly enlarged the binding pocket (Figure 2C). Furthermore, the binding pocket is strictly conserved; only two of the CSP5 binding-residues have been mutated in the seven ant species and even these have not changed into amino acids with different chemical properties (see Table 2, Dataset S2 and Table S1). The opposite trend is observed in ant CSP6 and 7. Several binding-residues in these proteins have mutated into larger ones (Table 2, Table S1), which appears to result in a shrunken binding pocket (Figure 2D). For example, there are following size increasing changes in CSP7 compared to PbarCSP1: F29W, L51F, L64F (Figure 2). The size distribution of binding residues is significantly different between the CSP7 (smallest pocket) and CSP5 (largest pocket) in all ant species except *L. humile* (Chi-squared test, P -values <0.05).

Moreover, CSP6 and 7 - also, to some extent, CSP3 - show greater variation between ant species in the binding-residues compared to the other orthologous ant CSPs (Table 2, Dataset S2 and Table S1). For example, the small, hydrophobic binding-residue L44 (numbering of the reference protein PbarCSP1), conserved in most CSPs, has mutated into a large aromatic amino acid, F, in HsalCSP6 and LhumCSP6. Two otherwise conserved hydrophobic binding-residues, L51 and L64, have become F in AechCSP6 and AcepCSP6. Furthermore, H62 is F in SinvCSP6, while it is a small-sized I in CSP6 of the other six species. Added to the great surface charge variation of CSP6-7 (Table 1), the binding pocket variation further suggests these orthologous proteins could function in a slightly different task in different ant species.

In summary, the orthologous ant CSPs (CSP1-7) differ in their size, charge and ligand-binding pocket properties. Ant CSP1-4 represent typical insect CSPs by their charge distribution and binding pocket structure. The C-terminal last helix has been modified and become shorter in CSP4 and 7, and CSP5 lacks it completely. The binding pocket appears to be adaptable for versatile ligands; the pocket is enlarged in the CSP5 model and reduced in CSP6-7. The charges and the binding pocket are highly conserved in CSP5 between the ant species, whereas variation in both surface charge and the binding pocket is enhanced in CSP6-7. Based on the structural characteristics, the orthologous ant CSPs can be grouped in the following way: typical CSPs (CSP1-4), 5-helical CSP (CSP5) and CSPs with smaller binding pocket (CSP6-7) (Figure 1).

### Genes Duplicated in Ants have Retained the Structural Characteristics of CSP7

**Figure 3:**
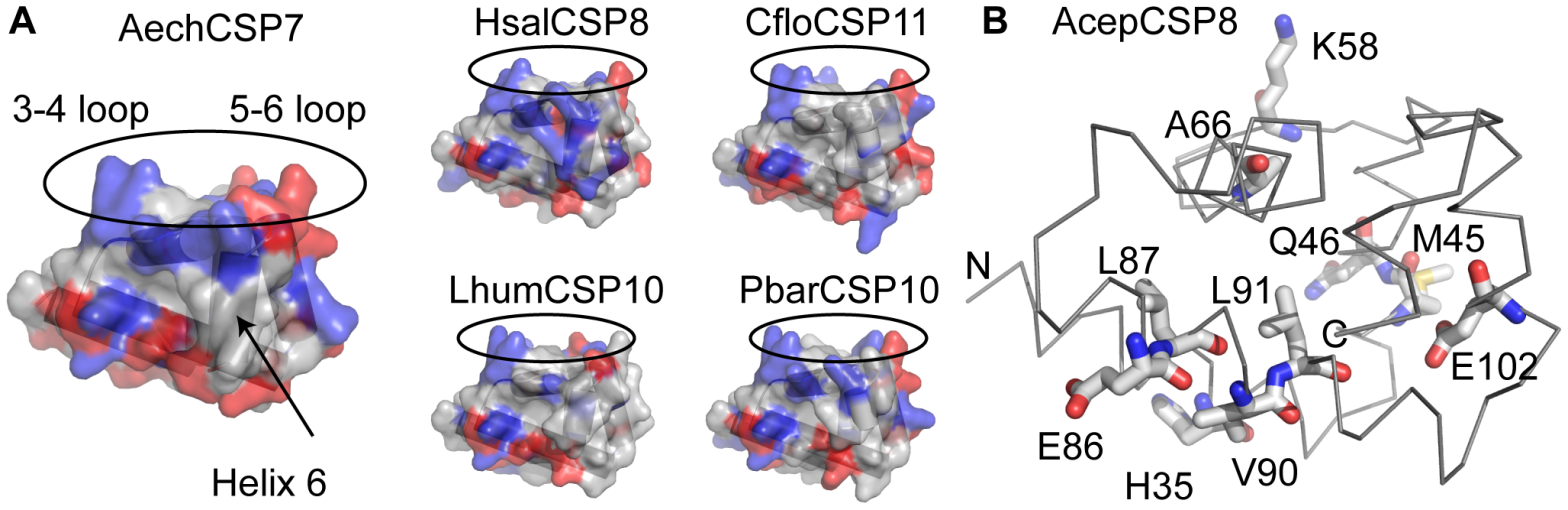
Ant-specific CSPs share similarities with CSP7, and positive selection in them is concentrated on the surface. (A) Left, molecular model of CSP7 shows a “crown” of charged residues (circled). The crown is formed by the positive loop between helices 3 and 4, and by the negative charge between helices 5 and 6. The ant-specific proteins (right) all have this crown. Positively charged residues (K, R) are shown in blue and negatively charged (D, E) in red. (B) The ten residues under positive selection (shown as sticks on the peptide backbone) mostly map on the surface. L87 and L91 are the only binding residues under positive selection. K58 and A66 are located near the “crown” and have various combinations of positive charge and hydrophobicity in the ant-specific CSPs. The N and C termini are indicated.

Next, we inspected representative proteins within the ant-specific CSP expansion. Phylogenetically, this expansion is grouped with the conserved ant gene CSP7, suggesting that the ant-specific expansion has originated from an ancestral ant CSP7 or a gene similar to this [8]. The phylogenetic relatedness of these proteins to CSP7 is further supported by their structural properties; the proteins within the ant-specific expansion have a truncated helix 6 (Figure 1) and similarities to CSP7 in their surface charges (Figure 3., “the crown”). The modeled ant-specific CSPs have sequence identity of 28–33% compared to the template structure. The Ramachandran assessment of the models was good (>90% in the favored region and <5% in the outlier region), except for the lower values of CfloCSP11 (91.6% and 5.3%) and LhumCSP10 (92.6% and 8.3%).

The ant-specific CSPs we modeled, and also the majority of those we did not model (found in the Dataset S2) have a conserved positive charge in the loop between helices 3–4 (K76, K78, K79 in PbarCSP10) and a conserved negative charge of 1–2 amino acid residues in the loop between helices 5–6 (E112 in PbarCSP10). These conserved amino acid residues create a charge pattern of a “crown” (Figure 3A). Loop 3–4 is likely very rigid, due to a cysteine bridge in that location, and might be an important structural element for these proteins. Otherwise, the charges of the 57 solvent accessible residues (criteria: >20% solvent accessible amino acid in at least four of the seven modeled ant-specific proteins) vary in over half of the cases (32 residues). An additional similarity between the ant-specific expansion and CSP7 is that these proteins have many large amino acids in their binding pocket, likely resulting in a small pocket (Table S1). Despite the similarities, these proteins might have different ligand preferences due to the differences in their binding-residues (Table S1). For example, the binding-residue corresponding to PbarCSP1 V48 is large Y or F in LhumCSP10, PbarCSP10 and AcepCSP8 but smaller I in CfloCSP11. Another example is L64, which has mutated into amino acids of large size in all the above mentioned proteins except in PbarCSP10 (L).

Previous results [8] have shown signatures of positive selection in the ant-specific expansion of CSPs. Fourteen amino acid sites were indicated to be under positive selection, of which ten were included in the modeled sequence and eight of these are solvent-facing residues (Figure 3B, Table 3). The modeled proteins had a total of 65 solvent-facing amino acid sites. Charge variation was present in all eight solvent-facing positively selected sites and in 32 of the remaining 57 sites. There is, thus, significantly higher charge variation in the positively selected surface residues than in the rest of the surface residues (Fisher’s exact test, P = 0.0465). Especially interesting are the two positively selected residues that are spatially close to the “crown” (A66 and K58 in AcepCSP8 numbering) and their purpose could be to increase or reduce the charge of the conserved crown. The two non-solvent accessible positively selected residues, L87 and L91, are binding-residues. Variation in these could have direct consequences on ligand-binding.

**Table 3:** Chemical characteristics of the ten amino acid residues under positive selection.

Aa under positive selection (AcepCSP8 numbering)	Average solvent accessible area (standard deviation)	Binding residue?	Aa variation (charged residues underlined)
H35	59.16% (14.09)	No	ILHD
M45	45.63% (18.69)	No	LTIMNQERKD
Q46	35.90% (13.6)	No	GQSE
K58	65.32% (28.68)	No	VILPTMVKRD
A66	33.19% (17.59)	No	APTQNSHKR
E86	52.23% (13.82)	No	ILVNYQHKED
L87	3.10% (2.02)	Yes	ILMQNYHR
V90	34.79% (17.62)	No	ALVIFTMQYSHREDK
L91	9.11% (3.29)	Yes	LIVAMT
E102	22.29% (7.48)	No	AIMTQNEKRD

The residue numbering is according to AcepCSP8. The average solvent accessible area (with standard deviation) was calculated for the seven modeled ant-specific CSPs, and is indicated for each positively selected residue. Binding residues correspond to the residues at 5 Å distance from the ligands in MbraCSPA6 structure. The amino acid variation for each positively selected residue is shown in the last column, where all the ant-specific proteins of the seven species were taken into account. All solvent accessible positively selected amino acid residues show charge variation that can potentially modify the proteins’ electrochemical surface interactions.

Dataset S2 Alignment used in homology modeling and phylogeny reconstruction.

In conclusion, CSPs within the ant-specific expansion are similar in size, have partial similarities in their charge (the crown) and have larger binding-residues than typical CSPs. All these properties are shared by the suggested ancestral protein, CSP7. Thus, the ligands bound by these proteins are likely to be different from those bound by CSP1-5 and more similar to CSP6 and CSP7 instead. However, CSPs within the ant-specific expansion do have several variable sites indicated to be under positive selection [8]. These sites are located mostly on the surface, but one of them points directly into the binding pocket. Positively selected sites on the protein surface have extensive variation in charge, suggesting positive selection has driven surface charge changes in the ant-specific CSP expansion.

### Diversification of Solenopsis Invicta Duplicates

The fire ant *S. invicta* has the highest number of CSP genes among the studied ant species and the largest *S. invicta* expansion constitutes eight fast-evolving CSP genes [8], including the putative nestmate cue binder and one pseudogene [16]. We examined this expansion in more detail by modeling all the functional genes within it, namely CSPSinv13, 15–17 and 19–21 (Figure 1). There is a relatively low, but sufficient sequence identity, 22–26%, between the SinvCSPs and the template structure. A sequence-based secondary structure prediction was performed on SinvCSP15 that, exceptionally, had only 18% identity. The sequence-based structure prediction supported the structure found with homology modeling (Dataset S1). The Ramachandran assessment was excellent for SinvCSP13, SinvCSP15, SinvCSP19, SinvCSP20 and SinvCSP21 (>95% of residues in favored region and <1% in the outlier region), but lower for two models: SinvCSP17 (88% and 7.2%) and SinvCSP16 (90.5% and 8.3%).

The modeled *S. invicta* CSPs have completely lost helix 6, resembling in that respect the conserved 5-helical ant CSP5. The *S. invicta* CSPs do not resemble each other greatly in regards to their surface charge, with the exception of the hydrophobicity of the C-terminal side (Figure 1), another feature common with the ant CSP5. However, the hydrophobic patch is not as large as the one in CSP5 (Figure 1). The binding-residues have mostly size-increasing and charge-changing mutations of the same order of magnitude as the other ant-specific CSPs (Table S1). Exceptions to this are two conserved large binding-residues (F29 and Y47 in PbarCSP1) that have been mutated into smaller ones (L, I, and A, V, I, E, respectively) in *S. invicta* CSPs 13, 15 and 19–20, which is an additional similarity to CSP5 (Table S1). Similar to CSP6, CSP7 and the other ant-specific CSPs, the *S. invicta* proteins have variation in their binding-residues; for example, binding-residue V48 (PbarCSP1 numbering) is found as V, L, I, D or E in these proteins (Table S1). The ancestral CSP7 or a similar protein has been duplicated several times in *S. invicta* and each duplicate has been modified in novel ways regarding both surface charge and binding-residues.

### Arthropod CSP Phylogeny

**Figure 4:**
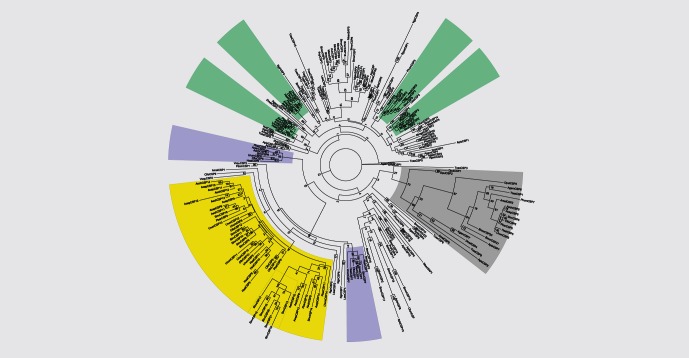
Arthropod CSP phylogeny. Maximum likelihood tree constructed from representative arthropod CSP protein sequences. Species are referred to in three letter codes. Confidence values (1000 bootstraps) are indicated. Different CSP groups are highlighted in different colors. Typical CSPs are highlighted in green and CSPs with smaller binding pocket in purple. All 5-helical CSPs in arthropods form a single clade which is highlighted in grey. The ant-specific expansion is highlighted in yellow.

We built a phylogeny of arthropod CSP proteins jointly with the modeled protein structures to infer how different CSP structures are distributed within the arthropods. The phylogeny includes the complete set of CSPs from the seven ant species and species representing different orders within the Arthropoda; including the insect groups Lepidoptera, Hymenoptera, Orthoptera, Coleoptera, Diptera, Hemiptera, Phthiraptera as well as one species from each of Crustacea and Chelicerata (Figure 4) Overall, the deep branches have little bootstrap support suggesting that CSPs from different insect orders (and beyond) have diverged so much that there is little similarity between them, making phylogeny reconstruction difficult. Generally speaking, CSPs group together by order or species suggesting that these sequences have duplicated and differentiated after those insect orders have diverged.

Only one of the modeled ant CSPs (CSP5) forms a well supported orthologous group with genes outside Hymenoptera. The clade containing this 5-helical ant CSP5 contains genes from nearly all included Arthropoda with a reasonable bootstrap support (73%) (Figure 4). Further support to the group is given by the fact that all these orthologous proteins are 5-helical, lacking the last helix, as opposed to the typical six α-helices of insect CSPs (Dataset S2). Some species, like *T. castaneum*, *A. mellifera*, *A. pisum* and *A. gambiae* have two 5-helical CSPs whereas ants, *D. melanogaster* and *P. humanus* seem to have independently lost one of the copies.

CSP phylogeny also allows insight into the early evolution of arthropod CSPs. The crustacean *D. pulex*, which diverged from insects over 450 Mya, has only three CSPs and all of them are 5-helical and belong to the conserved clade of 5-helical CSPs (Figure 4). The Chelicerata (*I. scapularis)* diverged from other taxa used here roughly 700 Mya and has a single 6-helical CSP. This suggests both 5- and 6-helical CSPs have been present early in arthropod evolution. The majority of the present insect CSPs are 6-helical, and have duplicated and diversified during the evolution of the insect orders.

## Discussion

Although CSPs are seemingly conserved six helical ligand-carrying proteins, they can be extensively modified by evolution. We show how the size, surface charges and binding pocket of ant CSPs have evolved in these proteins. Based on our combined work on protein modeling and phylogenetics, the ant CSPs can be divided into three groups; 1) typical CSPs, 2) a highly conserved 5-helical CSP that has orthologous copies in nearly all the studied arthropod taxa and 3) hymenopteran or ant-specific CSPs with smaller binding pockets.

### 1) Typical CSPs are 6-helical and have Conserved Binding Pockets, but Variable Surface Charge

Ant CSP1-4 are identified here as typical CSPs, since they resemble each other and several other insect CSPs such as MbraCSPA6 by their evenly distributed, but variable surface charges and their highly similar binding pockets. Although these proteins differ from each other in surface charge, the variation between ant species is minimal, with the exception of CSP3 that is more variable. The conservation of CSP1-2 and 4 suggests that the function of at least these CSPs is similar in all ant species. It appears that CSPs 1–4 might carry ligands that have relatively similar chemical properties, since their ligand-binding residues are well-conserved. The known CSP ligands of the orthologous honey bee proteins include aliphatic alcohols, esters, amides and aromatic compounds [14], [28], [31]. Apart from the *A. mellifera*, *Nasonia* and a few *T. castaneum* genes, there are no reasonably supported orthologs for ant CSP1-4 in other species. This either could mean that these genes are mostly Hymenoptera specific or that orthologous genes from other orders have diverged so much that orthologs cannot be identified.

### 2) 5-helical CSP5 is Structurally and Phylogenetically Unique among CSPs

The five helical CSP5 is unique in its small size and surface hydrophobicity. The binding-residues are smaller compared to other CSPs, suggesting this protein might bind larger ligands. This is supported by the honeybee CSP binding data showing that the orthologous AmelCSP2 binds large aromatic molecules [14]. Increased hydrophobicity is connected to decreased solubility [32]. Thus, we speculate that the diffusion rate and/or tissue environment might be different for CSP5 compared to the more charged CSPs. Speculation is limited by the low sequence identity (19%) of CSP5 to the template structure, which easily results in errors in homology modeling [29]. However, our CSP5 model was supported by purely sequence-based structural prediction and the Ramachandran assessment for CSP5 was excellent, which increases confidence in our model.

The structural uniqueness of CSP5 is accompanied by its phylogenetic conservation (Figure 4). Our large phylogenetic comparison identified CSP5 as an ancient CSP present in all the taxa in which the complete set of CSPs have been annotated, the sole exception being in the tick *I. scapularis*. We suggest that the role of this particular CSP is different from other CSPs. CSP5 is unlikely to perform chemosensory functions for several reasons. First, the arthropod chemosensory systems and chemical signals are diverse. Therefore, CSPs involved in chemosensation are expected to be multifaceted and lack highly similar orthologs in arthropod species. CSP5, conversely, has extremely conserved orthologs in ants (estimated dN/dS = 0.09 in [8]) and in other arthropods. Furthermore, functional and expression data support the conclusion that these proteins are involved in processes other than chemosensation. In the honeybee, one of the 5-helical proteins (AmelCSP5) has been identified as a regulator of embryonic development [10] and it is expressed only in the ovaries and eggs. The other 5-helical CSP in *A. mellifera* (AmelCSP2) is ubiquitously expressed and shows low levels of expression in nearly all examined tissues, life stages and castes [13]. Furthermore, the crustacean *D. pulex* has three CSPs and all of them are 5-helical. It is the only species of those included here still living in an aquatic environment and thus has different chemosensory requirements compared to other arthropod species. Taken together, the current data implies that the 5-helical CSPs do not function in chemosensation, but rather have a ubiquitous role in development or an important housekeeping function and that this function is conserved across arthropods.

### 3) Ant-specific CSPs have Evolved Extensive Variation on the Surface and a Smaller Binding Pocket

Ant CSP6, CSP7 and the ant-specific CSP expansion have generally larger ligand-binding residues than ant CSP1-5. Many of these amino acid residues become completely buried in the binding pocket with no surface access upon ligand binding based on MbraCSPA6 structure [26]. The large binding-residues could result in these CSPs binding smaller and/or less branched ligands or smaller amounts of ligand than CSP1-5. On the other hand, these CSPs (except CSP6) have a C-terminal truncation, which might provide better access for some ligands to the inner cavity.

CSPs similar to ant CSP7 seem to be Hymenoptera specific on the basis of our CSP phylogeny (Figure 4.). In *Camponotus japonicus,* CSP7 binds the cuticular hydrocarbons used in nestmate recognition and is the major protein expressed in the antennae [3]. Proteins orthologous or similar to the *C. japonicus* protein are also expressed in the antennae in *L. humile* (LhumCSP7) [17], *A. mellifera* (AmelCSP1) [13] and *P. dominulus* (PdomCSP1) [33]. Furthermore, the *A. mellifera* CSP1 and the *P. dominulus* CSP1 have similar binding preferences [14], [31]. AmelCSP1 binding preference for straight chain primary alcohols and esters clearly decreases when the compound size exceeds 14 carbon atoms [14]. Also, PdomCSP1 seems to prefer alcohols and amides with carbon chain length 14–16 [31]. Because of similar expression and structural conservation of these proteins, we suggest that CSP7 binds similar substances, including cuticular hydrocarbons, in Hymenoptera. The ant-specific CSP expansion has been derived from CSP7 type of ancestor and may share these functions. This is supported by the fact that all the modeled proteins within the ant-specific expansion have partially retained the surface charge and the binding pocket size changing mutations of the ant CSP7. Furthermore, the ant-specific duplicates appear to keep one location of charge; this is the so called crown, which is also found in CSP7.

Summarizing, ant CSP6-7 and CSPs within the ant-specific expansion clearly differ from other modeled ant CSPs and their structural conservation suggests they could bind similar ligands, including cuticular hydrocarbons among others. In addition to their role in nestmate recognition, ants use cuticular hydrocarbons as an information source to distinguish workers that perform different tasks [34]. Furthermore, they are used to indicate fertility and dominance status [35]. Thus the expansion and diversification of ant CSPs could reflect the variety of information content in cuticular hydrocarbons and their importance in ant chemical communication.

We inspected more closely one branch of ant-specific duplicates, the largest *S. invicta* specific expansion (Figure 4). This expansion was shown to evolve fast and putatively under positive selection (dN/dS = 1.2 as estimated in [8]) and to contain a possible nestmate cue binder of *S. invicta* [16]. These *S. invicta* CSPs share similarities with CSP6, CSP7 and duplicates in other ant species in that they all have mostly large binding-residues of varying charge. The *S. invicta* CSPs also appear to have clear signatures of their own; these proteins have fully lost the last helix and have increased surface hydrophobicity and in this respect resemble the conserved CSP5. The charge of *S. invicta* CSPs is highly variable and does not obey the charged crown pattern of other CSPs in the ant-specific expansion. Presumably, these *S. invicta* CSP duplicates have adapted for individual ligand and/or receptor-binding. This is supported by the work of González et al. [16], who show that one of these proteins, the putative nestmate cue binder, does not bind cuticular hydrocarbons, but instead binds polar cuticular lipids such as fatty acids and esters.

### Targets of Positive Selection

The earlier analyses indicated that CSPs within the ant-specific expansion have evolved under positive selection [8]. Two of the positively selected sites are among the binding-residues, but all the remaining positively selected sites are surface residues. This finding suggests that natural selection has favored the diversification of the surface residues rather than changes in the binding pocket. This is in contrast to proteins that function in similar tasks as CSPs, namely the OBPs, where positive selection was suggested to predominantly act on binding residues [36]. The surface sites in CSPs have extensive variation in charge, which is significantly higher than the overall charge variation of the surface, further suggesting positive selection is driving surface charge changes. The extensive variation in surface charge in the ant CSP family seems therefore to be an adaptation associated with the function of CSPs in a similar way as suggested, for example, in the case of *Pgi* duplication in teleost fishes [37]. In that case, different *Pgi* copies are expressed tissue specifically, and have evolved differing surface charges through weak positive selection on several amino acid sites. Similar findings have also been made in other duplicated genes [38].

Another chemosensory gene family, the OR family, has expanded to include hundreds of copies in ants [39], [40]. Generally, it has been thought that different ORs bind different odors and this is the first discriminatory step in olfaction [4], while CSPs and OBPs act as general carriers with wide binding spectra. Now, growing evidence shows that some CSPs and OBPs can bind selectively [14], [41], with drastic conformational changes taking place upon ligand binding [26], [42]. Campancci et al. [26] hypothesized that conformational changes in CSPs, depending on which ligand has been bound, could direct the CSP to different ORs. Taking this into account, we suggest that the positively selected sites on the surface and the resulting charge variation of CSPs could either affect the specificity of the CSP to a certain ligand or mediate interaction between the CSP and specific ORs. Alternatively, surface charge variation might be linked to the cellular environment where the CSP is expressed, as in the case of *Pgi* in teleost fishes [36]. The role of the conserved charged crown suggests it is important for the ant-specific CSPs and could also be important in OR binding or ligand recognition.

### Origin and Evolution of CSPs within Arthropods

One of the most ancient arthropod CSPs was 5-helical, as suggested by the conservation of the 5-helical protein from crustacea to modern insects. However, both 5- and 6-helical CSPs were present early in Arthropod evolution and currently it is impossible to say which one of those is the ancestral form. Available functional data suggests the structure and function of CSPs are linked; the 5- helical proteins function in conserved processes not related to chemosensation, and the 6-helical CSPs have duplicated and diversified and perform chemosensory functions, among others. No clear orthologs, except for the 5-helical CSP, can be distinguished between insect orders. Available data, although limited, suggests orthologous CSPs can be found within insect orders and these possibly have conserved functions.

### Conclusions

Here we combined protein modeling and phylogenetic analysis to study the structure and evolution of CSPs, demonstrating the advantages of using comparative modeling in evolutionary context. Our ant examples show that CSP proteins have extensive size, charge and binding pocket variation that can presumably be linked to their interaction with ORs or ligands. The group of CSPs that have expanded in ants are likely involved in chemosensation. Interestingly, positive selection in these ant-specific duplicates has driven changes in the surface charge of these proteins, further suggesting adaptive significance of surface charge in CSPs. Both modeling and phylogenetics highlighted a group of unique CSPs, whose conserved, ancient 5-helical structure is distinct from other CSPs and likely involved in functions other than chemosensation. While phylogenetic and molecular evolutionary analysis can reveal how proteins diverge and which evolutionary forces drive their evolution, modeling can give insight into which properties of the proteins are being modified in evolution.

## Materials and Methods

To study the structural variation in CSPs, we built homology models of several ant CSPs. As modeling is based on an accurate multiple sequence alignment, we built a multi-taxa alignment using MAFFT [43] using the G-INS-i method. In addition to template and modeled amino acid sequences we included all the CSPs from the seven ant species (*Camponotus floridanus* (Cflo), *Harpegnathos saltator* (Hsal), *Solenopsis invicta* (Sinv), *Linepithema humile* (Lhum), *Pogonomyrmex barbatus* (Pbar), *Acromyrmex echinatior* (Aech) and *Atta cephalotes* (Acep)) and several other insect taxa; *Apis mellifera*, *Bombyx mori*, *Tribolium castaneum*, *Anopheles gambiae*, *Drosophila melanogaster, Acyrthosiphon pisum*, *Pediculus humanus*, *Daphnia pulex* and *Ixodes scapularis*. In addition to these we used hymenopteran sequences from [33], [44] and Lepidopteran sequences from [45] as well as CSPs from *Plutella xylostella*, *Amyelois transitella* and *Megoura viciae* (Dataset S2.). We used the same alignment to produce a CSP arthropod phylogeny with 1000 bootstrap replicates using maximum likelihood in RAxML [46], [47].

As a modeling template we used the non-ligand bound structure MbraCSPA6 (PDB-ID:1KX9) from *M. brassicae*. With the exception of the last helix, CSPs contain ultraconserved residues evenly spread (approximately every fifth residue apart) in the sequence, for example, (PbarCSP1 numbering) Y25, D30, I37, R42, Y47, D53, D60, K65, A71, Q83, I90, L94, W102 and Y109 (see Dataset S2 for sequence alignment). Notably, all CSPs have four cysteine residues spread out in the sequence that build two cysteine bridges. These cysteine bridges, the evenly spread conserved residues, the lack of long insertions or deletions and the sequence based secondary structure predictions (see Dataset S1) suggest that the tertiary structure is likely highly similar in most CSPs. Thus, CSPs make reliable modeling targets. We chose representative sequences for modeling in the seven ant species. The sequences for modeling were chosen based on a minimal number of insertions or deletions compared to the template sequence and minimal sequence variation within the group of CSPs to be modeled. The modeled proteins of the conserved orthologous ant CSP genes were: CSP1-CSP6 of *P. barbatus* and CSP7 of *A. echinatior*. The identity scores between the modeled CSPs and the template were calculated in ClustalW [48]. Additional amino acid sequence-based secondary structure prediction was performed in the case of the two CSPs that had sequence identity below 20% (PbarCSP5 and SinvCSP15) using the software PSIPRED [48]. This approach does not rely on any existing structure, but is based on sequences that are associated with secondary structures (helices, β-sheets and coils). Thus, the prediction offers a way to estimate the reliability of a model. We compared the protein models of orthologous ant CSPs to infer structural differences and similarities between them. We also studied the extent of variation of each CSP between ant species. In order to compare the orthologous ant CSPs to the CSPs in the ant-specific expansion, we modeled a subset of proteins from the ant-specific expansion. One protein from each ant species was chosen from different parts of the gene phylogeny in order to cover variation within the ant-specific expansion. The modeled proteins were HsalCSP8, AcepCSP8, CfloCSP11, LhumCSP10, PbarCSP10 and AechCSP14 and seven proteins in a *S. invicta* specific expansion (SinvCSP13, Sinv15-17, Sinv19-21). Modeling was done with homology modeling software Bodil [49] that enables rapid model construction based on a homologous template structure when the number of insertions and deletions is low (as in the case of CSPs). This program uses the peptide backbone of the template structure to create a model, adjusts the backbone torsion angles, and adds the amino acid side chains based on torsion angles. An alignment of the template structure and all the modeled ant CSPs (total 20) is in Dataset S3. Program RAMPAGE [30] was used to validate the peptide backbone chemistry, i.e., for Ramachandran assessment, which indicates the percentage of the model amino acids having chemically favored dihedral angle values. Generally, >90% of the amino acid residues should be at the favored region in the Ramachandran assessment for a reliable model [50].

The solvent accessible residues were calculated with GETAREA [51] with 20% solvent accessibility threshold. For the binding pocket size comparisons, binding residues were categorized into small (S,T,C,G,P,A and V), intermediate (H,D,E,N,Q,I and L) and large (R, K, F, Y and W). The illustrations were produced with the molecular visualization software PyMol (Version 1.2r3pre, Schrödinger, LLC), whose rotamer library was used to adjust the conformation of possible colliding residues at the binding pockets of the models.

The amino acid residues under positive selection were obtained from [8]. In AcepCSP8 sequence these residues are: H35, M45, Q46, K58, A66, E86, L87, V90, L91 and E102.

To compare the binding pockets, we defined the amino acid residues that are likely to interact with the ligands. We defined as ligand-binding residues those 34 residues that were at atomic interaction distance (5 Å) from the three bromo-dodecanol molecules in the ligand-bound *M. brassicae* CSPA6 structure (Figure 1A). These residues were: Y24, D25, I27, L29, I32, L39, Y42, V43, V46, E55, G56, E58, L59, H62, L63, A66, I67, G70, C71, C74, N77, Q78, G81, A82, V85, I86, L89, W97, L100, T101, D105, W110, R111 and Y114 in MbraCSPA6. For Table S1., these residues are named as the corresponding amino acids of PbarCSP1 based on the sequence alignment (Dataset S2). The sequence alignment was also the basis of comparison of charged residues in CSPs. Here, D, E, K and R were considered charged residues.

## Supporting Information

Table S1 Variation in the ligand-binding residues of ant CSPs.
(PDF)

Dataset S1 Secondary structures of CSP5 and SinvCSP15 by homology modeling and sequence prediction (PSIPRED).
(DOC)

Dataset S2 Alignment used in homology modeling and phylogeny reconstruction.
(FASTA)

Dataset S3 The modeling template sequence (MbraCSPA6; PDB-ID: 1KX9) and the modeled ant CSP sequences.
(DOC)
