# Supplementary material for: Insights into the Evolution of the CSP Gene Family through the Integration of Evolutionary Analysis and Comparative Protein Modeling
Source: PLoS One. 2013 May 28;8(5):e63688. doi: 10.1371/journal.pone.0063688 (PMC3665776; doi:10.1371/journal.pone.0063688)
Supplement: Table S1 — Variation in the ligand-binding residues of ant CSPs. (PDF) [file pone.0063688.s001.pdf]

Binding residues (PbarCSP1 numbering)

		F29	V32	V34	I37	L44	Y47	V48	L51	D60	A61
Typical CSPs	CSP1										
	CSP2										
	CSP3								2xM		
	CSP4										
5-helical	CSP5	V	E e		A		Q		A	V e	
Smaller pocket	CSP6					2xF e		2xM	2xF		
	CSP7			P		R e		Y	FIV		Q
	Ant-sp.					R e		YFR e	YF	EDVAS e	ALMQ
	SinvCSP	LIYF				R e	AVIES e	LVIDE e	YFI	EQLV e	EQK e

		E63	L64	N67	L68	A71	I72	D75	A82	Q83	A86
Typical CSPs	CSP1			N					ATM		AM
	CSP2			TIAHS					K		G
	CSP3			HYVHNV					RKH		IVLM
	CSP4			T				GNSD e	K		MT
5-helical	CSP5	R e		L				S e	E	E e	Q
Smaller pocket	CSP6	SYT- e	2xF	IF		2xM		STKI e	KR		TMGMI
	CSP7	FY e	FML	HN			F	K e	TMVR		NG
	Ant-sp.	FT e	FYL	MIFTK	FAVIYM		FMIY	KRQN e	K		MHLNR
	SinvCSP	YKRL e		ILAKS	FI			NDK e	KT	2xR e	HNILCQ

		A87	I90	S91	L94	L105	E106	D110	Y115	R116	Y119
Typical CSPs	CSP1	A		S			E			R	
	CSP2	TS		IV			K			TSTI	
	CSP3	VAITS		IL			K			ATVS	
	CSP4	AT		I			AVMI			KR	
5-helical	CSP5	I		L			V	AVG e	-	-	-
Smaller pocket	CSP6	A		ISG		F	LVI		YHLRD e	VCMIV	FYIL
	CSP7	YVLM		VI	Y	ML	VIM	LMI e	KN e	LMQ	TPS-
	Ant-sp.	MHLNR	ILVM	VISQAE	YF	ILF	VLI	LVIQS e	KE e	KA	GQS-
	SinvCSP	ILVM		T	YF		LIV	DNHR e	-	-	-

Amino acid decreased in size
Amino acid increased in size
e Charge in electric change
Highly variable amino acid

The following conserved residues were not included in the table: D30, C76, C79 and W102.
