# Supplementary material for: Insights into the Evolution of the CSP Gene Family through the Integration of Evolutionary Analysis and Comparative Protein Modeling
Source: PLoS One. 2013 May 28;8(5):e63688. doi: 10.1371/journal.pone.0063688 (PMC3665776; doi:10.1371/journal.pone.0063688)
Supplement: Dataset S1 — Secondary structures of CSP5 and SinvCSP15 by homology modeling and sequence prediction (PSIPRED). (DOC) [file pone.0063688.s002.doc]

**PbarCSP5**

Helix1 G R S R V S D E Q L N M A L S

Model C H H H H H H H H H H H H H H

PSIPRED C C C C C C H H H H H H H H C

Conf 9 8 8 6 6 6 2 4 3 9 9 9 7 4 6

Helix2 D K R Y L N R Q L K C A

Model C H H H H H H H H H H H

PSIPRED C H H H H H H H H C C C

Conf 8 9 9 9 9 9 9 8 5 1 0 4

Helix3 L G E A P C D P V G R R L K S L V P L V L R G

Model C C C C C C C H H H H H H H H H H H H H H H H

PSIPRED C C C C C C C H H H H H H H H H H H H H H H C

Conf 3 7 8 9 8 8 8 7 8 8 8 8 8 8 7 5 7 7 9 9 8 2 5

Helix4 S C P Q C N P E E T R Q I K K V L S H I Q R S

Model C C C C C C H H H H H H H H H H H H H H H H H

PSIPRED C C C C C C H H H H H H H H H H H H H H H H H

Conf 7 9 9 9 9 9 9 9 9 9 9 9 9 9 9 9 9 9 9 9 9 7 1

Helix5 F P K E W N R I V Q Q Y A G V P

Model C H H H H H H H H H H H C C C C

PSIPRED C H H H H H H H H H H H C C C C

Conf 9 6 9 9 9 9 9 9 9 9 8 5 7 7 9 9

**SinvCSP15**

Helix1 S E L D D L D I P K I L A

Model C H H H H H H H H H H H H

PSIPRED C C C C C C C H H H H H C

Conf 9 8 8 6 6 8 8 8 8 8 7 5 4

Helix2 N D A E R Q G V I D C I

Model C H H H H H H H H H H H

PSIPRED C H H H H H H H H H H H

Conf 9 9 9 8 8 7 8 8 7 2 4 5

Helix3 L E N A S C T E L E T K A A A A I K D A L K T

Model C C C C C C C H H H H H H H H H H H H H H H H

PSIPRED C C C C C C C H H H H H H H H H H H H H H H H

Conf 0 5 9 8 8 8 8 8 9 9 9 9 9 9 9 9 9 9 9 9 9 6 1

Helix4 N C Q A C G D K R K E N M K I I T D W F N Q N

Model C C C C C C H H H H H H H H H H H H H H H H H

PSIPRED C C C C C C H H H H H H H H H H H H H H H H C

Conf 1 8 6 5 7 8 9 9 9 9 9 9 9 9 9 9 9 9 9 9 8 6 4

Helix5 Q P D T W T L V V A K V N S

Model C H H H H H H H H H H H C C

PSIPRED C H H H H H H H H H H H C C

Conf 9 2 7 8 9 9 9 9 9 7 4 1 8 9

**Supporting Dataset S1. Secondary structures of CSP5 and SinvCSP15 by homology modeling and sequence prediction (PSIPRED).** Two distinct approaches, modeling based on homology to MbraCSPA6 and sequence-based secondary structure prediction both assign CSP5 and SinvCSP15 proteins 5-helical. ‘Model’ shows the secondary structures present in the model (H=helix, C=coil). ‘PSIPRED’ shows the secondary structure prediction and ‘Conf’ is a confidence score (0-9; 9 is the highest value) for each secondary structure, calculated by the program PSIPRED. Red color highlights the matching helices of the model and the prediction, while pink highlights the non-matching helices.
