# Supplementary material for: Insights into the Evolution of the CSP Gene Family through the Integration of Evolutionary Analysis and Comparative Protein Modeling
Source: PLoS One. 2013 May 28;8(5):e63688. doi: 10.1371/journal.pone.0063688 (PMC3665776; doi:10.1371/journal.pone.0063688)
Supplement: Dataset S3 — The modeling template sequence (MbraCSPA6; PDB-ID: 1KX9) and the modeled ant CSP sequences. (DOC) [file pone.0063688.s004.doc]

**Supporting Dataset S3. The modeling template sequence (MbraCSPA6; PDB-ID: 1KX9) and the modeled ant CSP sequences.**
